# Genetic Diversity of Methicillin‐Resistant *Staphylococcus aureus* Isolates From Two Tertiary Care Hospitals in Sulaymaniyah, Iraq, Characterized by *spa* Typing, Coagulase VNTR Sequencing, and REP‐PCR

**DOI:** 10.1155/ijm/9366780

**Published:** 2026-04-20

**Authors:** Alan O. Shwan, Sahand K. Arif

**Affiliations:** ^1^ Department of Biology, University of Sulaimani, Sulaymaniyah, 46001, Iraq, univsul.edu.iq; ^2^ Laboratory Department, Hiwa Hospital, Sulaymaniyah, 46001, Iraq

**Keywords:** coagulase (*coa*) VNTR, genetic heterogeneity, genotyping, MRSA, Simpson’s index of diversity

## Abstract

**Background:**

Methicillin‐resistant *Staphylococcus aureus* remains a major cause of health care‐associated infection in high‐risk units such as burn and cancer wards. In settings where advanced genotyping schemes are not routinely available, combining complementary molecular typing methods can improve characterization of genetic diversity for surveillance purposes.

**Methods:**

Over a nine‐month period, 54 nonduplicate MRSA isolates were collected from a burn hospital (*n* = 30) and a cancer hospital (*n* = 24) in Sulaymaniyah, Iraq. Species identity and methicillin resistance were confirmed by *nuc* and *mecA* PCR. Isolates were characterized using *spa* sequencing, sequence‐based coagulase (*coa*) VNTR typing and REP‐PCR fingerprinting. Discriminatory power was quantified using Simpson’s index of diversity (SID), and intermethod concordance was assessed using the adjusted Rand index and adjusted Wallace coefficients.

**Results:**

*spa* typing identified 16 types, dominated by t037 and t304 (SID = 0.884). Sequence analysis of the *coa* VNTR region revealed 38 distinct nucleotide repeat units encoding 20 amino acid motifs, resolving 10 composite *coa* types (SID = 0.839). REP‐PCR generated 36 fingerprint patterns grouped into 15 clusters, yielding the highest numerical discrimination (SID = 0.921). Concordance was moderate between *spa* and *coa* typing but low between REP‐PCR and either sequence‐based method, indicating substantial short‐range genetic heterogeneity (distinct REP‐PCR fingerprints). Shared *spa*/*coa* types across both hospitals indicate the presence of common genetic backgrounds, while heterogeneous REP‐PCR fingerprints within this cohort highlight fine‐scale genetic heterogeneity.

**Conclusions:**

MRSA isolates from these hospitals were dominated by a limited number of *spa* types and *coa* VNTR variants, with substantial fine‐scale genetic heterogeneity. *spa* typing provided a portable lineage‐background framework, sequence‐based *coa* VNTR analysis added reproducible, complementary resolution at a virulence‐associated locus, and REP‐PCR improved short‐range discrimination when interpreted alongside sequence markers, supporting pragmatic surveillance of MRSA genetic diversity. This work adds a sequence‐resolved *coa* VNTR repeat catalog and a quantitative cross‐method analysis to guide selection and interpretation of complementary MRSA typing schemes.

## 1. Introduction

Methicillin‐resistant *Staphylococcus aureus* (MRSA) remains a major contributor to health care‐associated infections worldwide and imposes a substantial clinical and economic burden on hospitals [1]. MRSA infections are associated with prolonged hospitalization, increased mortality, and higher treatment costs, in part because resistance to commonly used β‐lactam antibiotics limits effective first‐line therapy [2]. These challenges are amplified in high‐risk units, such as burn and oncology wards, where extensive skin‐barrier disruption, immunosuppressive therapy, and repeated antibiotic exposure facilitate MRSA colonization and transmission [3, 4].

Understanding MRSA transmission dynamics in hospitals is therefore essential for guiding effective infection‐prevention strategies. Over the past 2 decades, molecular typing has become indispensable for examining the population structure and dissemination patterns of MRSA in clinical settings, evolving from established approaches, such as PFGE, MLST, *spa* typing, and SCC*mec* typing to whole‐genome sequencing (WGS) with core‐genome SNP analysis and cgMLST/wgMLST [5]. WGS provides the highest resolution for hospital transmission analysis and is increasingly being incorporated into molecular epidemiologic investigations [6]. However, routine implementation of WGS in many resource‐limited settings remains constrained by requirements for specialized laboratory infrastructure, bioinformatics expertise, and substantial computational capacity [7]. Consequently, conventional and intermediate‐resolution typing methods continue to retain practical value for surveillance, preliminary cluster assessment, and interhospital comparison. Among the affordable and operationally feasible approaches, *spa* sequencing, which targets polymorphisms in the X region of the *spa* gene, is widely used due to its reproducibility, simplicity, and globally standardized nomenclature [8]. However, single‐locus methods interrogate only one polymorphic target; therefore, their discriminatory power can be limited in low‐diversity backgrounds during local outbreaks, and isolates may be misclassified because of recombination and/or homoplasy. Therefore, researchers often combine complementary genotyping approaches that target additional variable genomic regions to improve resolution [5].

The staphylococcal coagulase gene (*coa*) encodes a secreted virulence factor composed of a short N‐terminal signal peptide followed by two conserved functional domains, D1 and D2, which bind and activate prothrombin to form staphylothrombin. These are followed by a moderately conserved central region and a variable number tandem repeat (VNTR) region at its 3′ end, consisting of multiple 81‐bp repeat units that each encode a 27‐amino‐acid peptide. Variation in the number and sequence of these repeats produces substantial size polymorphism among *coa* alleles and forms the basis of widely used PCR‐RFLP/VNTR profiling, whereas sequence‐based *coa* VNTR typing has been comparatively less explored [9]. Similarly, repetitive‐element palindromic PCR (REP‐PCR) generates genomic fingerprint patterns that are particularly useful for differentiating strains in localized epidemiological investigations [10]. Used together, these methods support the interpretation of genetic relatedness and epidemiologic linkage within healthcare facilities [11]. Despite their value, comparative analyses of *spa* sequencing, *coa* VNTR sequence typing, and REP‐PCR remain scarce in the Middle East, including Iraq, where MRSA continues to pose a significant public health challenge. The lack of integrated molecular epidemiology studies limits understanding of how MRSA circulates between high‐risk hospital departments, constraining surveillance and infection control efforts.

In this context, this study investigates the genetic diversity of MRSA from two high‐risk tertiary care settings in Sulaymaniyah, Iraq, using rapid, cost‐effective, and scalable typing approaches that capture both genetic background and fine‐scale genetic heterogeneity. We applied *spa* sequencing as a portable lineage‐background framework, coagulase VNTR sequencing to add virulence‐linked, sequence‐based resolution, and REP‐PCR as a genomic fingerprint for short‐range differentiation. Discriminatory power and intermethod agreement were quantified (SID, adjusted Rand index [ARI], and adjusted Wallace [AW] coefficients) to clarify what each method contributes to relatedness inference within and across hospitals. The main contribution of this study is an integrated characterization of *coa* VNTR sequence diversity, coupled with a formal, metrics‐based comparison of the applied genotyping approaches within the same MRSA cohort, supporting surveillance that distinguishes shared circulating genetic background (*spa*/*coa*) from finer‐scale differentiation captured by REP‐PCR.

## 2. Methods

### 2.1. Study Design and Setting

This study employed a cross‐sectional molecular typing design. Isolates were collected from two tertiary hospitals to capture MRSA populations arising from distinct clinical settings with different patient profiles and selective pressures, rather than to directly compare hospitals. The burn hospital and the cancer hospital represent contrasting high‐risk environments for MRSA colonization and infection, thereby providing a genetically heterogeneous isolate collection suitable for evaluating and comparing the discriminatory performance of molecular typing methods. In parallel, a descriptive molecular epidemiologic linkage analysis was performed by integrating composite genotype with the collection date and ward metadata.

### 2.2. Bacterial Isolates

A total of 54 nonduplicate MRSA isolates were collected over a nine‐month period (January 1, 2024, to September 30, 2024) from two tertiary hospitals in Sulaymaniyah, Iraq: a specialized burn hospital (*n* = 30) and a cancer hospital (*n* = 24). Isolates originated from clinical specimens obtained from admitted patients with suspected infection (blood, urine, sputum, burn wound, diabetic foot, surgical wound, vaginal swab, aspirated body fluids, and wound tissue) as well as screening skin and nasal swabs from healthcare personnel. For each isolate, the admitting/working ward at the time of sampling and the collection date were recorded. Only one isolate per patient or staff member was included to avoid duplication. Isolate IDs were prefixed by hospital: E = burn hospital; H = cancer hospital throughout the study. ATCC 6538 was used solely as a control, not included in diversity or epidemiologic analyses.

This study was reviewed and approved by the Academic and Scientific Ethics Committee, College of Science, University of Sulaimani. Patient specimens were obtained during routine diagnostic care with no additional procedures performed for research purposes. Healthcare worker nasal/skin swabs were collected as part of the hospital infection‐prevention program. The ethics committee approved the secondary use of de‐identified specimens/data for research and waived the requirement for individual informed consent. All data were coded, handled confidentially, and reported in aggregate. The study was conducted in accordance with the ethical principles of the World Medical Association Declaration of Helsinki [12].

### 2.3. Phenotypic Identification

Preliminary identification of *S. aureus* was conducted using routine microbiological methods, including mannitol fermentation, catalase testing, and tube coagulase assays, following established diagnostic procedures [13]. Species identification was confirmed using the BD Phoenix M50 (PMIC/ID‐111 panel) automated system (Becton Dickinson, USA).

### 2.4. Bacterial Genomic DNA Extraction

Genomic DNA was extracted from fresh overnight cultures using the AddPrep Bacterial Genomic DNA Extraction Kit (AddBio Inc., South Korea), following the manufacturer’s instructions. The concentration and purity (A260/A280 and A260/A230 ratios) of the extracted DNA were assessed using a Thermo Scientific NanoDrop spectrophotometer (Fisher Scientific).

### 2.5. Genotypic Confirmation of *S. aureus*

Species‐level confirmation was performed by PCR targeting the *nuc* gene, a species‐specific thermonuclease marker widely used for *S. aureus* identification [14, 15]. Primers used were *nuc*‐F: GCG​ATT​GAT​GGT​GAT​ACG​GTT and *nuc*‐R: AGC​CAA​GCC​TTG​ACG​AAC​TAA​AGC [14]. PCRs were carried out using the AddBio HotStart PCR Master Mix (AddBio Inc., South Korea) in a 25 μL reaction volume under the following cycling conditions: 95°C for 5 min; 35 cycles of 95°C for 20 s, 57°C for 20 s, and 72°C for 20 s; followed by a final extension at 72°C for 5 min and expected 279 bp amplicon. Amplicons were visualized using 1.5% agarose gel electrophoresis in 1 × TBE buffer. A representative target amplicon was purified using the AddPrep Gel Purification Kit (AddBio, South Korea) and subjected to Sanger sequencing using the same primers (Macrogen Inc., South Korea). In all PCR assays, *Staphylococcus aureus* ATCC 6538 served as the positive control, while nuclease‐free water was included as a nontemplate control in each run.

### 2.6. Determination of Methicillin Resistance

Preliminary screening for methicillin‐resistant phenotype was based on the BD Phoenix M50 susceptibility reports. All Phoenix‐flagged MRSA isolates were confirmed by cefoxitin (30 μg) disk diffusion in accordance with the CLSI M100, 34^th^ edition (Clinical and Laboratory Standards Institute) [16]. In addition, all isolates were screened for *mecA* and *mecC* genes using specific PCRs as a genotypic confirmation of the MRSA isolates [5]. Primers used for *mecA* were *mecA*‐F: GTA​GAA​ATG​ACT​GAA​CGT​CCG​ATA​A, *mecA*‐R: CCA​ATT​CCA​CAT​TGT​TTC​GGT​CTA​A [17], with a PCR cycling of 95°C for 5 min, followed by 35 cycles of 95°C for 30 s, 56°C for 30 s, and 72°C for 30 s, with a final extension at 72°C for 6 min and expected 310 bp amplicon. Primers used for *mecC* were as follows: *mecC*‐F: ACT​AGT​ATC​TCG​CCT​TGG, *mecC*‐R: ATCCCGAGTGATTATCCC [18], with PCR cycling of 95°C for 5 min, followed by 35 cycles of 95°C for 20 s, 53°C for 20 s, and 72°C for 20 s, with a final extension at 72°C for 5 min and expected 104 bp amplicon. All PCR amplicons were resolved by 1.5% agarose gel electrophoresis in 1 × TBE. A representative target amplicon was purified and subjected to Sanger sequencing using the same primers (Macrogen Inc., South Korea).

### 2.7. *spa* Typing

Amplification of the *spa* X region (3′ end repeat region) was performed using primers and cycling parameters recommended by the Ridom SpaServer protocol [19]. The following primer pair *spa*‐1113f: TAA​AGA​CGA​TCC​TTC​GGT​GAG​C and *spa*‐1514r: CAG​CAG​TAG​TGC​CGT​TTG​CTT was used under the following PCR cycling conditions: initial denaturation of 95°C for 5 min, followed by 35 cycles of 95°C for 45 s, 59°C for 45 s, and 72°C for 90 s, with a final extension at 72°C for 10 min. PCR amplicons were visualized using the QIAxcel Advanced System (Qiagen, Germany) with the QIAxcel DNA High Resolution Kit (Cat. No. 929004), following the manufacturer’s instructions. Purified products were subsequently subjected to Sanger sequencing using the same primers (Macrogen Inc., South Korea). Chromatograms were inspected and trimmed using Chromas software v2.2.6; then, sequences were assigned to *spa* types using Ridom SeqSphere + v10.5.4, following established nomenclature rules. Final *spa* sequences attributed to each isolate including ATCC 6538 were submitted to the NCBI GenBank database.

### 2.8. *coa* (Staphylococcal Coagulase) Gene VNTR Typing

**FIGURE 1:**
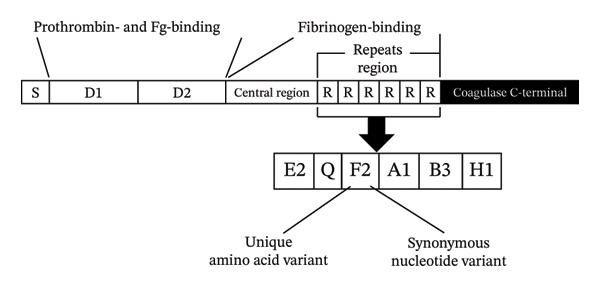
Domain structure of *coa* gene including the signal peptide (S), D1‐D2 prothrombin/fibrinogen‐binding domains, the central region, and the variable 81‐bp tandem repeat (R) region, followed by the conserved C‐terminal domain. The repeat‐region sequence was used to generate alphanumeric profiles for *coa* repeat typing.

The VNTR region at the 3′ end of the *coa* (staphylococcal coagulase) gene was amplified using the primer pair *coa*‐F: ATA​GAG​ATG​CTG​GTA​CAG​G and *coa*‐R: GCT​TCC​GAT​TGT​TCG​ATG​C [20], under the following PCR condition: initial denaturation at 95°C for 5 min, followed by 35 cycles of 95°C for 30 s, 52°C for 45 s, and 72°C for 60 s, with a final extension at 72°C for 10 min. PCR amplicons were visualized using the QIAxcel Advanced System (Qiagen, Germany) with the QIAxcel DNA High Resolution Kit (Cat. No. 929004). Purified amplicons were subsequently submitted for Sanger sequencing using the same primers to Macrogen Inc. (South Korea). Raw sequences were trimmed using Chromas software v2.2.6 and screened for tandem repeats using Tandem Repeats Finder [21]. A *coa* repeat‐region reference sequence (GenBank accession no. AB488507) was used for alignment and repeat‐boundary confirmation [22]. Repeat arrays were analyzed to classify both nucleotide‐level repeat units and their corresponding amino acid sequences (81‐bp repeat units encoding 27‐amino‐acid repeats). Unique amino acid repeat motifs were designated using uppercase letters, whereas distinct nucleotide variants encoding the same amino acid repeat were differentiated using numeric suffixes (e.g., A1, A2, B1, and B2) only when multiple synonymous variants were observed. These combined repeat signatures were used to generate *coa* repeat profiles (Figure 1), and isolates sharing identical profiles were assigned to the same *coa* type. This typification strategy was adapted from Shopsin et al. [22], with study‐specific modifications described above. Final *coa* sequences attributed to each isolate were submitted to the NCBI GenBank database.

### 2.9. REP‐PCR Fingerprinting

The repetitive extragenic palindromic (REP) region was amplified by REP‐PCR using the single primer RW3A (5′‐TCG​CTC​AAA​ACA​ACG​ACA​CC‐3′) [23]. PCR was performed with an initial denaturation at 95°C for 5 min, followed by 35 cycles of 95°C for 45 s, 54°C for 45 s, and 72°C for 90 s, with a final extension at 72°C for 10 min. PCR amplicons were screened using the QIAxcel DNA High Resolution Kit (Cat. No. 929004) to ensure high‐resolution fragment analysis. Electropherograms were exported for analysis using GelJ software [24] to perform band‐based clustering, employing the Dice similarity coefficient and the UPGMA clustering algorithm with a 1.0% band‐matching tolerance. REP‐PCR clustering was defined using a ≥ 90% similarity cutoff that was selected a priori and applied consistently across the dataset.

### 2.10. Data and Statistical Analysis

The discriminatory ability of each typing method (*spa* sequencing, *coa* VNTR sequencing, and REP‐PCR) was assessed using the Simpson’s index of diversity (SID; Hunter–Gaston discriminatory index) with 95% confidence intervals (CI) and confidence intervals of numerical agreement (CINA) [25]. To determine the degree of agreement between typing schemes, the ARI for overall concordance and AW coefficients for directional predictive power between methods, with 95% CIs, were calculated [26].

All discriminatory and concordance statistics were computed using the Comparing Partitions online tool (http://www.comparingpartitions.info/index.php?link=Tool accessed on August 20, 2025), which implements the algorithms described by Carriço et al. [26] and Severiano et al. [27]. The tool was used to generate partition similarity matrices, bootstrap‐derived CIs, and pairwise comparisons between typing methods. Results were interpreted following established guidelines for evaluating typing system performance [28].

### 2.11. Descriptive Molecular Epidemiologic Linkage Analysis

To support epidemiological description, a composite “triple‐profile” was defined as the exact combination of *spa* type + *coa* type + REP cluster. Isolates sharing identical triple profiles were tabulated within each hospital and across hospitals. For each repeated triple profile, the temporal span (first to last detection) and the minimum interisolate interval (days) were calculated descriptively. Repeated profiles were additionally categorized by ward concordance and collection interval as high (same ward ≤ 7 days), moderate (same ward 8–30 days), or low (otherwise), using a “same ward within a week” contact concept applied in MRSA transmission/contact analyses [29], with the 8–30 days of stratum included as a study‐defined extension to capture less immediate ward‐local recurrence. These temporal and ward‐based categories were applied as descriptive heuristics to structure recurrence patterns and do not represent validated thresholds for inferring transmission.

## 3. Results

### 3.1. MRSA Isolates

All 54 isolates obtained from the burn hospital (*n* = 30) and the cancer hospital (*n* = 24) were confirmed as *S. aureus* through amplification of the *nuc* gene, yielding the expected 279 bp PCR product with the deposited accession no. of PX118930 in the NCBI GenBank for the representative sequence. Phenotypically, all isolates classified as MRSA based on BD Phoenix M50 and cefoxitin disk diffusion susceptibility testing were fully concordant with genotypic results. PCR analysis confirmed that every isolate harbored the *mecA* gene, producing the expected 310 bp amplicon (GenBank accession no. PX118931). In contrast, *mecC* was not detected in any isolate, indicating the absence of this alternative methicillin‐resistant determinant in the studied MRSA population.

### 3.2. *spa* Typing

**FIGURE 2:**
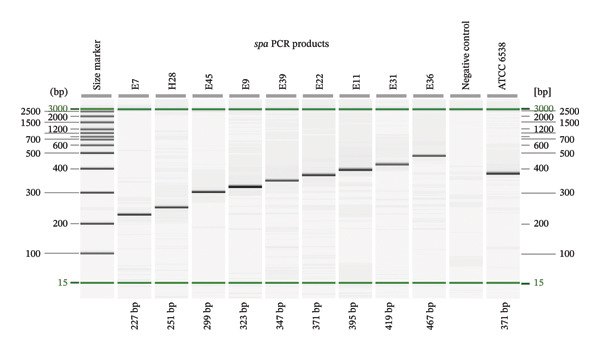
Capillary gel electrophoresis profiles of *spa* PCR products from representative MRSA isolates (E7–E36), generated using the QIAxcel Advanced System (Qiagen). Amplicons ranged from 227 to 467 bp and resolved into nine distinct size classes. A size marker is shown on the left, with a negative control and *Staphylococcus aureus* ATCC 6538 included for quality control.

**FIGURE 3:**
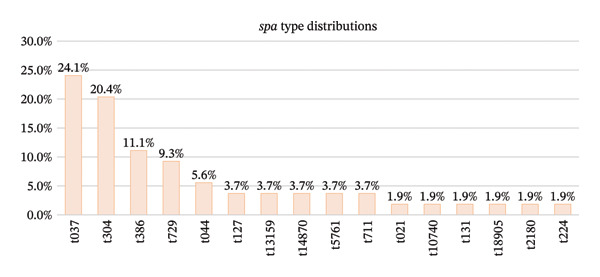
Bar chart summarizing the frequency of *spa* types in the pooled dataset. The data demonstrate a heterogeneous *spa* type distribution dominated by two major types (t037 and t304), with multiple sporadic types present at low frequencies, reflecting substantial clonal diversity within the MRSA population sampled from both hospitals.

All tested isolates (*n* = 54) successfully amplified the *spa* X region, producing PCR amplicons in nine different sizes ranging from 227 bp to 467 bp (Figure 2) with the GenBank accession numbers of PV926129‐PV926143 and PV990202‐PV990241 (*n* = 55 sequences: 54 clinical isolates plus the reference strain *S. aureus* ATCC 6538). Sequence analysis using the Ridom SpaServer identified a total of 16 distinct *spa* types among the isolates. The most prevalent *spa* types were t037 (*n* = 13) and t304 (*n* = 11), together accounting for nearly half of all isolates. These were followed by t386 (*n* = 6) and t729 (*n* = 5). Several types occurred at lower frequencies, including t044 (*n* = 3) and a group of *spa* types detected twice each: t13159, t14870, t711, t5761, and t127. Six *spa* types were represented by a single isolate: t224, t131, t021, t18905, t10740, and t2180 (Figure 3).

### 3.3. Discriminatory Power of *spa* Typing

Analysis of *spa* types revealed substantial allelic diversity within each hospital and across the pooled dataset. In the burn hospital (*n* = 30), 11 distinct *spa* types were identified (t037, t729, t044, t304, t711, t5761, t2180, t021, t224, t131, and t386), yielding a SID of 0.841. In the cancer hospital (*n* = 24), 9 *spa* types were detected (t304, t386, t037, t14870, t13159, t127, t18905, t10740, and t729), with a slightly higher SID of 0.848. When isolates from both hospitals were analyzed together (pooled dataset, *n* = 54), the number of partitions increased to 16 *spa* types, while 4 types were shared between the two hospitals (t037, t304, t386, and t729), resulting in an overall SID of 0.884 (Table 1).

**TABLE 1:** Summary of *spa* type diversity among MRSA isolates from each hospital and the pooled cohort.

Hospital	*N* isolates	# partitions (*spa* types)	SID	CI (95%)	CINA (95%)
Burn	30	11	0.841	(0.739–0.944)	(0.734–0.949)
Cancer	24	9	0.848	(0.753–0.943)	(0.744–0.952)
Pooled	54	16	0.884	(0.837–0.931)	(0.834–0.934)

*Note:* Values include the number of unique *spa* partitions, Simpson’s index of diversity (SID), and 95% confidence intervals (CI and CINA).

### 3.4. *coa* VNTR Typing

**FIGURE 4:**
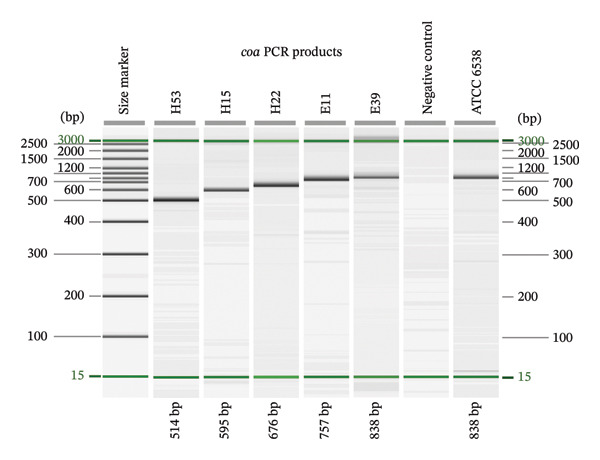
Capillary gel electrophoresis profiles of PCR amplicons corresponding to the VNTR region at 3′ of the *coa* gene from representative MRSA isolates (H53–E39), analyzed using the QIAxcel Advanced System (Qiagen). Amplicons ranged from 514 to 838 bp and grouped into five discrete size classes. A size marker, negative control, and *Staphylococcus aureus* ATCC 6538 are shown.

**FIGURE 5:**
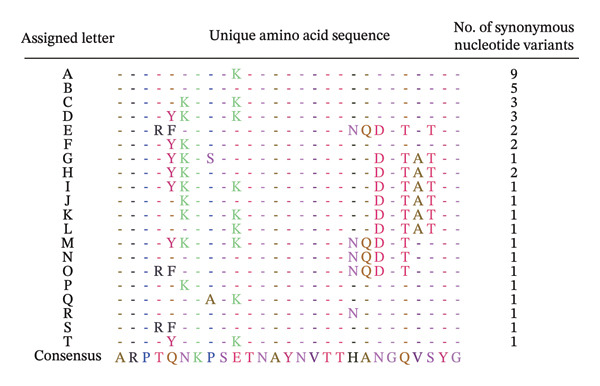
The 20 unique 27‐amino‐acid repeat motifs identified in the 3′ variable region of the *S. aureus coa* gene, annotated with the number of corresponding synonymous nucleotide variants observed in this study. Amino acid residues identical to the consensus sequence are indicated by dashes.

**FIGURE 6:**
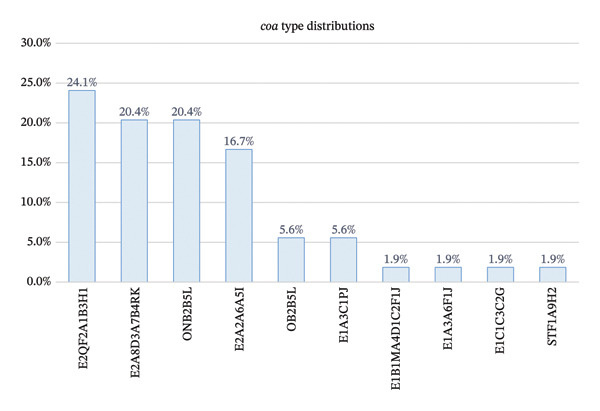
Bar chart illustrating the proportional distribution of staphylococcal *coa* types identified in the study cohort. The data demonstrated substantial allelic diversity, with multiple repeat combinations giving rise to a wide range of *coa* genotypes.

All 54 MRSA isolates successfully amplified the VNTR region of the *coa* gene, generating amplicons of five different sizes ranging from 514 to 838 bp (Figure 4) with the GenBank accession numbers of PX113510‐PX113520, PX277305‐PX277335, and PX452470‐PX452482. Sequence analysis revealed 38 unique 81‐nucleotide repeat units (Supporting Table S1) corresponding to 20 unique 27‐amino‐acid repeat motifs (Figure 5). These repeat elements, in various combinations and orders (4–8 repeat units per isolate), produced 10 distinct *coa* types across the isolate collection. The most common types were E2QF2A1B3H1 (*n* = 13), E2A8D3A7B4RK (*n* = 11), and ONB2B5L (*n* = 11), followed by E2A2A6A5I (*n* = 9). Less frequent types included E1A3C1PJ (*n* = 3) and OB2B5L (*n* = 3). Four types (STF1A9H2, E1A3A6F1J, E1B1MA4D1C2F1J, and E1C1C3C2G) were each represented by a single isolate (Figure 6).

### 3.5. Discriminatory Power of *coa* VNTR Typing

The *coa* VNTR locus demonstrated high discriminatory capacity across isolates from both hospitals. Among isolates from the burn hospital (*n* = 30), nine distinct *coa* types were identified (ONB2B5L, E2A8D3A7B4RK, E2QF2A1B3H1, E1A3C1PJ, E2A2A6A5I, OB2B5L, E1C1C3C2G, E1B1MA4D1C2F1J, and E1A3A6F1J), yielding a SID of 0.816. In the cancer hospital (*n* = 24), five *coa* types were detected (E2QF2A1B3H1, E2A2A6A5I, E2A8D3A7B4RK, OB2B5L, and STF1A9H2), corresponding to a SID of 0.754, revealing an acceptable discriminatory strength, though slightly lower than that observed in the burn hospital, reflecting the more limited allelic diversity in this subset.

When all isolates were analyzed together (pooled, *n* = 54), the number of partitions increased to ten *coa* types with four types shared between the two hospitals (E2QF2A1B3H1, E2A2A6A5I, E2A8D3A7B4RK, and OB2B5L), resulting in a SID of 0.839 (Table 2).

**TABLE 2:** Summary of *coa* VNTR diversity among MRSA isolates from the burn hospital, cancer hospital, and the combined dataset.

Hospital	*N* isolates	# partitions (*coa* types)	SID	CI (95%)	CINA (95%)
Burn	30	9	0.816	(0.722–0.910)	(0.716–0.916)
Cancer	24	5	0.754	(0.672–0.836)	(0.657–0.850)
Pooled	54	10	0.839	(0.802–0.877)	(0.797–0.882)

*Note:* Values include the number of distinct *coa* types identified, Simpson’s index of diversity (SID), and 95% confidence intervals (CI and CINA).

### 3.6. REP‐PCR

**FIGURE 7:**
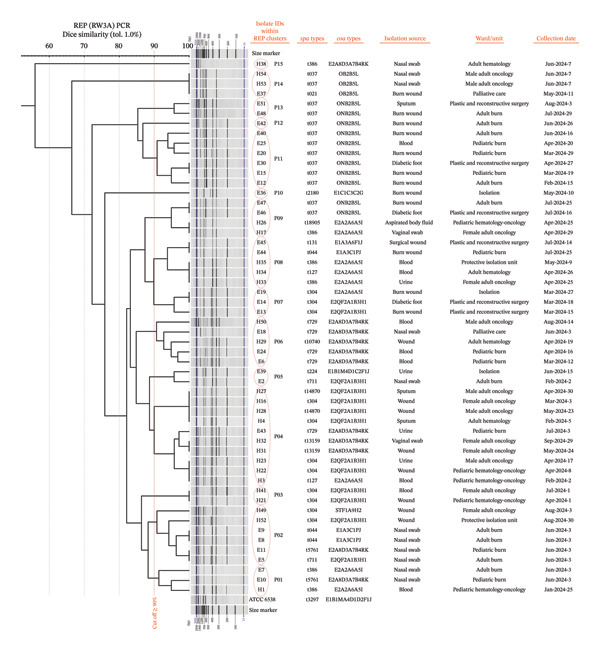
Capillary gel electrophoresis profiles of *S. aureus* isolates generated by REP‐PCR using the RW3A primer, alongside the corresponding UPGMA dendrogram based on Dice similarity (tolerance 1%). Clustering was defined at a ≥ 90% similarity cutoff. Isolate IDs are shown within their respective REP clusters in circles (P01–P15), with matched *spa* and *coa* types displayed for each isolate. Isolate IDs are shown in H or E letters with numeric suffix (1 to 54 each for an isolate): H stands for the cancer hospital and E for the burn hospital.

REP‐PCR produced heterogeneous genomic fingerprinting patterns (36 distinct REP patterns) across the 54 MRSA isolates. Banding profiles ranged from 2 to 10 distinct amplicons per isolate, with estimated fragment sizes ranging from 200 bp to 2500 bp. Clustering analysis resolved the isolates into 15 clusters, designated P01‐P15 (Figure 7). Among these, P04 was the most frequent cluster (10 isolates, 18.5%), followed by P02 and P11 (6 isolates each, 11.1%). Intermediate frequency clusters included the following: P06 and P08 (5 isolates each, 9.3%), and P09 (4 isolates, 7.4%). Several clusters including P14, P01, and P07 were represented by three isolates each (5.6%): lower prevalence clusters, such as P13, P05, and P03 (2 isolates each, 3.7%), and rare singletons including P12, P10, and P15 (1 isolate each, 1.8%).

### 3.7. REP‐PCR Discriminatory Power

The discriminatory performance of REP‐PCR (RW3A), separately for each hospital and for the pooled dataset, was as follows: Among the burn hospital isolates (*n* = 30), REP‐PCR resolved the collection into 11 distinct REP clusters (Supporting Figure S1), yielding a SID of 0.901. The cancer isolates (*n* = 24) exhibited 7 REP clusters (Supporting Figure S2), with a slightly lower SID of 0.833. When all 54 isolates were analyzed together, the number of detected partitions increased to 15 REP clusters, with an SID of 0.921 (Table 3).

**TABLE 3:** Discriminatory performance of REP‐PCR (RW3A) among MRSA isolates recovered from a burn hospital, a cancer hospital, and the pooled dataset shows Simpson’s index of diversity (SID) and corresponding 95% confidence intervals (CI) and confidence interval of numerical agreement (CINA).

Hospital	*N* isolates	# partitions (REP clusters)	SID	CI (95%)	CINA (95%)
Burn	30	11	0.901	(0.851–0.951)	(0.842–0.960)
Cancer	24	7	0.833	(0.749–0.918)	(0.738–0.929)
Pooled	54	15	0.921	(0.894–0.949)	(0.890–0.952)

*Note:* Higher SID values indicate greater discriminatory capacity.

### 3.8. Congruence Analysis Between Typing Methods

**FIGURE 8:**
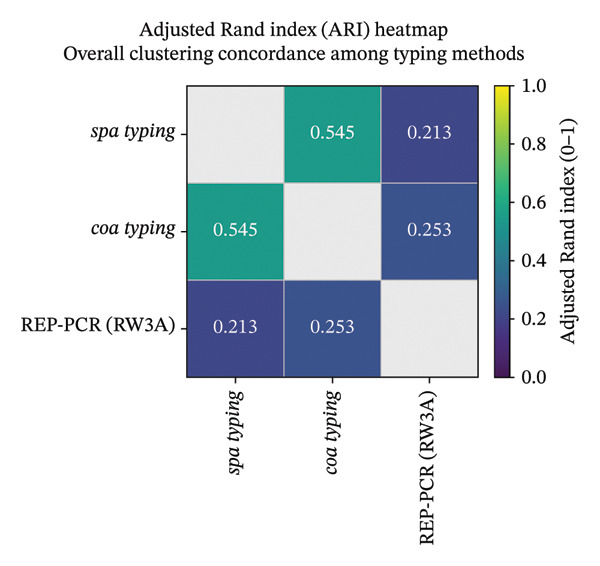
Heatmap of adjusted Rand indices summarizing overall clustering concordance among *spa* typing, *coa* VNTR typing, and REP‐PCR (RW3A). Values range from 0 (no agreement beyond chance) to 1 (perfect agreement), reflecting similarity between the classification outputs of each typing method.

Congruence among the three typing methods using the ARI indicated low to moderate concordance between methods. The highest ARI was observed between *coa* VNTR typing and *spa* typing (ARI = 0.545), suggesting partial overlap in the clustering structure defined by these two sequence‐based methods. In contrast, REP‐PCR exhibited only weak agreement with either *spa* typing (ARI = 0.213) or *coa* typing (ARI = 0.254) (Figure 8).

**FIGURE 9:**
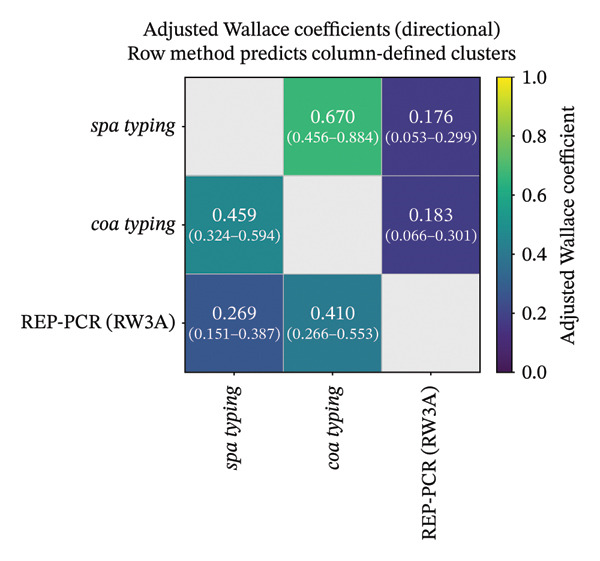
Heatmap of adjusted Wallace coefficients (with 95% bootstrap confidence intervals) quantifying directional predictive concordance among *spa* typing, *coa* VNTR typing, and REP‐PCR (RW3A). Values indicate the extent to which cluster assignments by the row method predict those defined by the column method (row ⟶ column); higher coefficients denote stronger directional agreement.

AW coefficients showed moderate but asymmetric directional concordance among methods. *spa* typing more strongly predicted *coa* clustering (AW = 0.67) than the reverse direction (*coa* ⟶ *spa* AW = 0.459). Prediction of REP‐PCR clustering from sequence‐based typing was weak (*spa* ⟶ REP‐PCR AW = 0.176; *coa* ⟶ REP‐PCR AW = 0.183), whereas prediction from REP‐PCR to sequence‐based schemes was higher, particularly REP‐PCR ⟶ *coa* (AW = 0.41) and REP‐PCR ⟶ *spa* (AW = 0.269) (Figure 9).

### 3.9. Descriptive Molecular Epidemiologic Linkage Findings

Repeated *spa*/*coa*/REP triple profiles were identified within each hospital and across both sites. In the cancer hospital, multiple repeated profiles were detected; most showed low ward/interval concordance, with a limited number meeting high or moderate concordance criteria. In the burn hospital, repeated profiles were likewise observed, including profiles meeting high concordance, while several others were categorized as low because detections were separated by ward and/or collection interval (Table 4). Across both hospitals, a small number of triple profiles were shared between institutions; all cross‐site recurrences were classified as low concordance under the predefined criteria (Table 5).

**TABLE 4:** Repeated *spa*/*coa*/REP triple profiles (≥ 2 isolates) identified within each hospital (H and E).

	*spa*/*coa*/REP‐profile	Isolates	First ⟶ last (span)	Min interval (d)	Ward/interval category
Cancer hospital (H)	t304/E2QF2A1B3H1/P04	H4, H16, H22, and H23	Feb 5, 2024 ⟶ Apr 17, 2024 (72 d)	9	Low (multiple wards)
	t304/E2QF2A1B3H1/P03	H21 and H41	Apr 1, 2024 ⟶ Jul 1, 2024 (91 d)	91	Low (multiple wards)
	t14870/E2QF2A1B3H1/P04	H27 and H28	Apr 30, 2024 ⟶ May 23, 2024 (23 d)	23	Moderate (same ward; 8–30 d)
	t13159/E2A8D3A7B4RK/P04	H31 and H32	May 24, 2024 ⟶ Sep 29, 2024 (128 d)	128	Low (same ward but > 30 d)
	t386/E2A2A6A5I/P08	H33 and H35	Apr 25, 2024 ⟶ May 9, 2024 (14 d)	14	Low (multiple wards)
	t037/OB2B5L/P14	H53 and H54	Jun 7, 2024 ⟶ Jun 7, 2024 (0 d)	0	High (same ward; ≤ 7 d)

Burn hospital (E)	t729/E2A8D3A7B4RK/P06	E6, E24, and E18	Mar 12, 2024 ⟶ Jun 3, 2024 (83 d)	35	Low (multiple wards)
	t044/E1A3C1PJ/P02	E8 and E9	Jun 3, 2024 ⟶ Jun 3, 2024 (0 d)	0	High (same ward; ≤ 7 d)
	t304/E2QF2A1B3H1/P07	E13 and E14	Mar 15, 2024 ⟶ Mar 18, 2024 (3 d)	3	High (same ward; ≤ 7 d)
	t037/ONB2B5L/P11	E12, E15, E20, E25, E30, and E40	Feb 15, 2024 ⟶ Jun 16, 2024 (122 d)	7	Low (multiple wards)
	t037/ONB2B5L/P09	E46 and E47	Jul 16, 2024 ⟶ Jul 25, 2024 (9 d)	9	Low (multiple wards)
	t037/ONB2B5L/P13	E48 and E51	Jul 29, 2024 ⟶ Aug 3, 2024 (5 d)	5	Low (multiple wards)

*Note:* For each profile, temporal span (first to last detection) and minimum interisolate interval (days) are summarized, and ward/interval concordance is classified as high (same ward ≤ 7 days), moderate (same ward 8–30 days), or low (otherwise).

**TABLE 5:** Shared *spa*/*coa*/REP triple profiles detected across both hospitals (H and E).

*spa*/*coa*/REP‐profile	Cancer hospital (H)	Burn hospital (E)	First ⟶ last (span)	Min interval (d)	Ward/interval category
t729/E2A8D3A7B4RK/P06	H50	E6, E24, and E18	Mar 12, 2024 ⟶ Aug 14, 2024 (155 d)	155	Low (multiple wards)
t386/E2A2A6A5I/P01	H1	E7	Jan 25, 2024 ⟶ Jun 3, 2024 (130 d)	130	Low (different wards/hospitals)

*Note:* For each cross‐site profile, temporal span, minimum interhospital interval (days), and ward/interval concordance category based on predefined criteria (high: same ward ≤ 7 days; moderate: same ward 8–30 days; and low: otherwise).

## 4. Discussion

This study provides a clear snapshot of MRSA genetic diversity circulating in two high‐risk hospitals within a single city, using three complementary typing methods. In addition to applying two well‐established approaches (*spa* sequencing and REP‐PCR), we characterized *coa* VNTR diversity by sequencing and performed a formal, metrics‐based comparison of discriminatory power and intermethod concordance across the three genotyping approaches within the same MRSA collection. Interpreting these findings alongside regional and international data helps clarify how “local” the circulating MRSA population is and provides practical guidance on how each method supports routine epidemiology in settings where WGS is not routinely available.

### 4.1. Confirmation of MRSA and *mec* Gene Profile

All 54 isolates were confirmed as *S. aureus* by *nuc* PCR and as MRSA by phenotypic cefoxitin screening and the presence of *mecA*, while none carried *mecC*. This pattern is entirely in line with global evidence that *mecA* remains the dominant methicillin‐resistant determinant, whereas *mecC* is still rare in human clinical isolates. Recent meta‐analyses and surveillance studies report *mecC* prevalences typically below 1% among MRSA from humans, even in countries where it is recognized, such as the United Kingdom and parts of Northern Europe [30, 31]. A recent local investigation has also reported the same pattern from the same hospital [32]. Other studies from the Middle East likewise show *mecA*‐positive/*mecC*‐negative MRSA profiles in hospital cohorts, including recent work from other Iraqi cities and neighboring countries [33–36]. In that sense, our findings fit a global pattern and suggest that, at least for now, routine *mecC* screening in this setting will primarily serve as a safeguard rather than a high‐yield diagnostic addition.

### 4.2. *spa* Typing and Population Structure

The overall SID for *spa* typing was high for the pooled dataset, with slightly lower but still robust values when burn and cancer hospitals were analyzed separately (Table 1). According to Hunter and Gaston’s criterion that a value > 0.90 is “highly discriminant,” our *spa* scheme falls just below that threshold but nevertheless offers substantial resolution.

The dominance of t037 and t304 is epidemiologically meaningful. Previous studies have shown that *spa* type t037 is frequently associated with classical health care‐associated MRSA clones, particularly the ST239/SCC*mec* III background, and has been reported as a major clone in several regions worldwide [37–42]. However, because *spa* typing is a single‐locus method, it should be interpreted as a surrogate marker of clonal background rather than a definitive clonal assignment, which is more reliably resolved by MLST together with SCC*mec* typing. Iraqi studies have also documented the recurrent presence of t037, t304, and t386 among clinical MRSA isolates, suggesting that these *spa* types are established in the region [43]. The emergence of less common types, such as t13159 and t14870, in our cohort echoes previous Iraqi work that identified novel or rare *spa* types in local hospitals, hinting at ongoing diversification and introduction of new allelic variants [44].

### 4.3. *coa* VNTR Diversity

Sequencing of the 3′ coding region of the *coa* gene, which contains the VNTR array, produced 10 *coa* types in the pooled collection, with four major types (E2QF2A1B3H1, E2A8D3A7B4RK, ONB2B5L, and E2A2A6A5I) accounting for most isolates. The pooled SID for *coa* typing was slightly lower than for *spa*, and diversity dropped further when hospitals were considered separately (Table 2). Although fewer studies have employed *coa* sequencing rather than RFLP, available work indicates that the *coa* VNTR region can offer good though often slightly lower discriminatory ability compared with *spa* typing [45]. Our SID values fit this pattern: *coa* clearly distinguished multiple allelic variants, but with somewhat broader clusters than *spa*. The repeat‐based classification framework used here, adapted from Shopsin and colleagues, (2000), but extended to separate amino acid motifs and their synonymous nucleotide variants, appears to capture fine‐scale repeat diversity while remaining interpretable at the level of “*coa* types.” This makes *coa* a useful complementary marker particularly when one wants to explore virulence‐associated loci in addition to the general clonal background.

### 4.4. Size‐Based Versus Sequence‐Based Resolution

Although capillary electrophoresis resolved *spa* and *coa* PCR products into a limited number of size classes, sequence‐based analysis revealed a substantially higher number of distinct *spa* and *coa* types, revealing that multiple types shared the same amplicon size. This discrepancy reflects the fact that PCR fragment length is determined primarily by the number of tandem repeat units only, whereas sequence‐based typing captures additional variation arising from differences in repeat composition, order, and synonymous nucleotide substitutions [8]. Consequently, isolates with identical amplicon sizes may represent distinct genetic types at the sequence level, which limits the discriminatory value of size‐based analysis alone and reinforces the necessity of sequencing for accurate *spa* and *coa* typing in molecular epidemiological studies.

### 4.5. REP‐PCR (RW3A) and Its Discrimination Performance

REP‐PCR using the RW3A primer generated complex banding profiles (2–10 bands, 200–2500 bp) consistent with the original RW3A rep‐PCR behavior reported by Del Vecchio et al. [23]. Dice/UPGMA clustering at a 90% similarity threshold resolved the isolates into 15 REP clusters. The pooled SID exceeded the 0.90 threshold, yielding the highest numerical discriminatory resolution among the three methods. Discriminatory power remained high when isolates from each hospital were analyzed separately (Table 3).

These values are broadly consistent with previous evaluations of RW3A‐based REP‐PCR, which have reported good reproducibility and strong discriminatory power for MRSA typing. Sabat et al. [10], for example, found that REP‐PCR approaches often rival PFGE in discrimination while being faster and more convenient for routine use. However, like other band‐based methods, REP‐PCR is sensitive to experimental conditions and can be less portable across laboratories, which partly explains why sequence‐based approaches, such as *spa* typing, have become the backbone of global surveillance.

### 4.6. Comparative Discriminatory Power and Congruence Between Methods

Across the three typing schemes, discriminatory performance was moderate to high, with REP‐PCR producing the highest SID, followed by *spa* and *coa* VNTR typing [25, 28]. Although these values fall below the 0.95 diversity benchmark that is often cited for a “highly discriminative” typing system, they still indicate useful differentiation for many applied epidemiologic purposes [28]. This ranking is consistent with the general expectation that genome‐wide fingerprinting approaches can yield finer partitioning than single‐locus sequence markers, while repeat‐based loci can show variable discrimination depending on locus diversity and analysis thresholds [10, 11]. Importantly, higher SID reflects finer partitioning of isolates but does not automatically translate into stronger evolutionary inference or better portability across laboratories [28].

Congruence metrics indicated that these methods captured overlapping but not equivalent signals of relatedness. The ARI results were compatible with moderate agreement between *spa* and *coa*, whereas agreement between REP‐PCR and either sequence‐based method was low. The AW results showed the expected directionality of prediction, reinforcing that the three methods are not interchangeable [27].

Biologically, partial *spa*‐*coa* concordance is plausible because both are single‐locus repeat/allelic markers that may track broader clonal structure to some degree, yet can diverge through locus‐specific processes (e.g., recombination at virulence loci, such as *coa*) [45]. By contrast, the low congruence between REP‐PCR and locus‐based types is consistent with comparisons showing that repetitive‐sequence PCR systems are most useful as rapid screening tools for short‐range outbreak investigations, may be less discriminatory than PFGE, and can be further subdivided by *spa* typing; accordingly, they should not be used as surrogates for portable lineage assignment without complementary sequence‐based data [11, 46, 47].

Overall, these results support treating REP‐PCR as a complementary, high‐resolution screen for short‐range clustering (e.g., ward‐level investigations), while *spa* and *coa* VNTR typing provide more portable, nomenclature‐driven outputs that are better suited to broader comparisons across time and place [11, 28].

From an infection‐prevention perspective, a practical stepwise approach in resource‐limited settings is to use *spa* ± *coa* to establish the circulating background, then apply REP‐PCR to prioritize short‐range investigations during suspected ward recurrences. Any fingerprint‐defined “cluster” should be interpreted alongside ward/time overlap and clinical context and treated as a trigger for investigation rather than standalone evidence of transmission [48].

### 4.7. Descriptive Molecular Epidemiologic Linkage Findings

Linking the composite genotype (*spa*/*coa*/REP) to collection time and ward location provides a pragmatic, rule‐based way to flag repeated occurrence patterns compatible with short‐range epidemiologic linkage [29]. Across both hospitals, repeated composite profiles were observed under multiple concordance categories, with many recurring patterns classified as low concordance because detections were separated by ward and/or longer time intervals. In high‐risk clinical environments, such recurrence is compatible with persistent circulation mediated by complex contact structures and indirect routes, including healthcare worker hands and the contaminated care environment [49]. However, these linkage outputs should be interpreted as hypothesis‐generating signals rather than proof of direct transmission. In particular, studies directly comparing conventional typing plus patient/ward overlap assumptions with WGS have shown that traditional approaches can both falsely infer transmission and miss true acquisition/transmission events, reinforcing the need for cautious inference [48].

At a broader population level, the similar diversity patterns observed with single‐locus markers, such as *spa* across hospitals, are compatible with both institutions sampling from a shared regional background of widely disseminated MRSA clonal complexes reported across the Middle East and North Africa [50]. In contrast, adding REP‐PCR to the composite genotype predictably reduces cross‐hospital profile matching by subdividing otherwise similar single‐locus backgrounds, consistent with the good resolving capacity of genome‐wide fingerprinting [11, 28].

### 4.8. Strengths of the Current Study

A key strength is the parallel application of three genotyping modalities alongside formal quantification of discrimination and between‐method congruence, which helps align method choice with the epidemiologic hypothesis [11, 28]. The findings provide actionable guidance: *spa* offers a practical balance of portability and discrimination; *coa* VNTR adds an additional locus‐level perspective; and REP‐PCR supplies a fine‐scale differentiation layer that is most defensible for short‐range clustering rather than lineage assignment [11, 46].

Methodologically, a notable contribution of this study is the use of *coa* repeat‐region sequencing to resolve variation at the nucleotide level rather than relying exclusively on *coa* PCR‐RFLP, which remains common in many settings [51]. Sequencing‐based characterization of *coa* alleles can clarify relationships among repeat‐region variants and highlight locus‐specific evolutionary processes [9, 45].

### 4.9. Limitations

This study is limited by a modest sample size, a single‐city setting, and a 9‐month sampling window, which may restrict generalizability. The absence of higher‐resolution genotyping (e.g., MLST or WGS) also limits definitive lineage confirmation and transmission inference, as previous studies show that WGS can refine or overturn conclusions based on conventional typing plus epidemiologic overlap alone [48]. Finally, adding SCC*mec* typing and/or WGS in future work would strengthen local cluster resolution and improve comparability of inferred strain structure across settings [5].

## 5. Conclusion

This study shows that MRSA isolates recovered from two tertiary hospitals in Sulaymaniyah, Iraq, are genetically heterogeneous when assessed using complementary typing methods. *spa* typing and sequence‐based *coa* VNTR analysis indicated predominant genetic backgrounds and broader population structure, while REP‐PCR offered fine‐scale differentiation among closely related isolates. However, REP‐PCR’s increased discrimination reflects improved local resolution rather than stronger phylogenetic inference. Taken together, the discrimination and congruence patterns indicate that these methods sample different layers of relatedness; therefore, their combined application provides a pragmatic framework for molecular characterization and surveillance in settings where advanced genotyping schemes are not routinely available.

## Funding

No funding was received for this manuscript.

## Disclosure

This research article forms part of a broader study conducted as a requirement of the authors’ doctoral (PhD) program. The work presented here represents an original component of that larger academic investigation.

## Ethics Statement

This study was reviewed and approved by the Academic and Scientific Ethics Committee, College of Science, University of Sulaimani (Approval No. 2281; June 23, 2023). The authors confirm that the study was conducted in accordance with the ethical principles of the World Medical Association Declaration of Helsinki for research involving human participants [12].

## Conflicts of Interest

The authors declare no conflicts of interest.

## Supporting Information

Additional supporting information can be found online in the Supporting Information section.

## Supporting information


**Supporting Information 1** Supporting Table S1: coa VNTR 81‐bp repeat‐unit nucleotide variants identified in this study, including assigned alphanumeric repeat‐unit codes and their corresponding 5′⟶3′ nucleotide sequences used to define composite sequence‐based coa VNTR types.

**Supporting Information 2** Supporting Figure S1: REP‐PCR (RW3A) fingerprinting dendrogram of MRSA isolates from the burn hospital (*n* = 30), showing clustering based on Dice similarity coefficients and UPGMA analysis.

**Supporting Information 3** Supporting Figure S2: REP‐PCR (RW3A) fingerprinting dendrogram of MRSA isolates from the cancer hospital (*n* = 24), showing clustering based on Dice similarity coefficients and UPGMA analysis.

## Data Availability

The data that support the findings of this study are available upon request from the corresponding author. The data are not publicly available due to privacy or ethical restrictions.
