# Supplementary material for: Genetic Diversity of Methicillin‐Resistant Staphylococcus aureus Isolates From Two Tertiary Care Hospitals in Sulaymaniyah, Iraq, Characterized by spa Typing, Coagulase VNTR Sequencing, and REP‐PCR
Source: Int J Microbiol. 2026 Apr 20;2026:9366780. doi: 10.1155/ijm/9366780 (PMC13094366; doi:10.1155/ijm/9366780)
Supplement: Supplementary file 1 — Supporting Information 1 Supporting Table S1: coa VNTR 81‐bp repeat‐unit nucleotide variants identified in this study, including assigned alphanumeric repeat‐unit codes and their corresponding 5′⟶3′ nucleotide sequences used to define composite sequence‐based coa VNTR types. [file IJM-2026-9366780-s001.docx]

| ***Alpha-Numeric Code*** | ***81-bp Repeat-Nucleotide Sequence (5′→3′)*** |
| --- | --- |
| **A1** | **GCCCGCCCAACACAAAACAAGCCAAGCAAAACAAATGCATACAACGTAACAACACATGCAAACGGCCAAGTATCATATGGC** |
| **A2** | **GCTCGCCCAACACAAAACAAACCAAGCAAAACAAATGCATACAACGTAACAACACATGCAAATGGTCAAGTATCATATGGC** |
| **A3** | **GCTCGCCCAACACAAAACAAGCCAAGCAAAACAAATGCATACAACGTAACAACACATGCAAATGGTCAAGTATCATATGGC** |
| **A4** | **GCTCGCCCAACACAAAACAAGCCAAGCAAAACAAATGCATATAACGTAACAACACATGCAAACGGCCAAGTATCATACGGA** |
| **A5** | **GCTCGCCCGACACAAAACAAGCCAAGCAAAACAAATGCATATAACGTAACAACACACGCAAACGGTCAAGTGTCATACGGA** |
| **A6** | **GCTCGCCCGACACAAAACAAGCCAAGCAAAACAAATGCATATAACGTAACAACACATGCAAATGGTCAAGTATCATACGGA** |
| **A7** | **GCTCGCCCGACACAAAACAAGCCAAGCAAGACAAACGCATATAACGTAACAACACATGCAAACGGCCAAGTATCATATGGC** |
| **A8** | **GCTCGTCCAACACAAAACAAGCCAAGTAAAACGAACGCATATAACGTAACAACACATGCAAATGGTCAAGTATCATACGGA** |
| **A9** | **GCTCGCCCGACACAAAACAAGCCAAGTAAAACAAATGCATATAACGTAACAACACATGCAAATGGTCAAGTATCATATGGA** |
| **B1** | **GCTCGCCCAACACAAAATAAGCCATCAGAAACAAATGCATATAACGTAACAACACATGCAAACGGCCAAGTGTCATACGGT** |
| **B2** | **GCTCGCCCGACACAAAACAAGCCAAGCGAAACAAACGCATATAACGTAACAACACATGCAAACGGCCAAGTATCATACGGA** |
| **B3** | **GCTCGCCCGACACAAAACAAGCCAAGCGAAACAAACGCATATAACGTAACAACACATGCAAATGGCCAAGTATCATACGGA** |
| **B4** | **GCTCGCCCGACACAAAACAAGCCAAGCGAAACAAATGCATATAACGTAACAACACATGCAAACGGTCAAGTATCATATGGC** |
| **B5** | **GCTCGTCCGACACAAAACAAGCCAAGCGAAACGAACGCATATAACGTAACAACACATGCAAACGGTCAAGTGTCATACGGA** |
| **C1** | **GCTCGCCCGACACAAAAAAAGCCAAGCAAAACAAATGCATACAACGTAACAACACATGCAAATGGTCAAGTATCATATGGC** |
| **C2** | **GCTCGCCCGACACAAAAAAAGCCAAGCAAAACAAATGCATATAACGTAACAACACATGCAAATGGTCAAGTATCATACGGA** |
| **C3** | **GCTCGCCCGACACAAAAAAAGCCAAGCAAAACAAATGCATATAACGTAACAACACATGCAAATGGTCAAGTATCATATGGC** |
| **D1** | **GCTCGCCCGACATACAAGAAACCAAGCAAAACAAATGCATACAACGTAACAACACATGCAAATGGTCAAGTATCATATGGC** |
| **D2** (ATCC 6538) | **GCTCGCCCGACATACAAGAAACCAAGCAAAACAAATGCATATAACGTAACAACACATGCAAATGGTCAAGTATCATACGGA** |
| **D3** | **GCTCGTCCGACATACAAGAAGCCAAGCAAAACAAATGCATACAACGTAACAACACATGCAAATGGTCAAGTATCATACGGA** |
| **E1** | **GCGAGACCAAGATTCAACAAGCCAAGTGAAACAAATGCATACAACGTAACGACAAATCAAGATGGCACAGTAACATATGGC** |
| **E2** | **GCGAGACCAAGATTCAATAAGCCATCAGAAACAAACGCATACAACGTAACGACAAATCAAGATGGCACAGTAACATATGGC** |
| **F1** | **GCTCGCCCGACATACAAGAAGCCAAGCGAAACAAATGCATACAACGTAACAACACATGCAAATGGTCAAGTATCATATGGC** |
| **F2** | **GCTCGTCCGACATACAAGAAGCCAAGCGAAACGAATGCATATAACGTAACAACACATGCAAACGGTCAAGTATCATATGGC** |
| **G** | **GCTCGCCCGACATACAAGAAGTCAAGCGAAACAAACGCATATAACGTAACAACACATGCAGATGGTACTGCGACATATGGG** |
| **H1** | **GCTCGCCCGACATACAAGAAGCCAAGCGAAACAAATGCATACAATGTAACAACACATGCAGATGGTACTGCGACATATGGG** |
| **H2** | **GCTCGTCCGACATACAAGAAGCCAAGCGAAACAAACGCATATAACGTAACAACACATGCAGATGGTACTGCGACATATGGG** |
| **I** | **GCTCGCCCGACATACAAGAAGCCAAGTAAAACAAATGCATACAATGTAACAACACATGCAGATGGTACTGCGACATATGGG** |
| **J** | **GCTCGCCCGACACAAAAAAAGCCAAGCGAAACAAACGCATATAACGTAACAACACATGCAGATGGTACTGCGACATATGGG** |
| **K** | **GCTCGCCCGACACAAAAAAAGCCAAGCAAAACAAATGCATATAACGTAACAACACATGCAGATGGTACTGCGACATATGGG** |
| **L** | **GCTCGCCCAACACAAAACAAGCCAAGTAAAACAAATGCATACAATGTAACAACACATGCAGATGGTACTGCGACATATGGT** |
| **M** | **GCTCGTCCGACATACAAGAAACCAAGTAAAACAAACGCATACAACGTAACGACAAATCAAGATGGCACAGTATCATATGGG** |
| **N** | **GCTCGCCCGACACAAAACAAACCAAGCGAAACAAACGCATACAACGTAACGACAAATCAAGATGGCACAGTATCATATGGC** |
| **O** | **GCGAGACCAAGATTCAACAAGCCAAGCGAAACAAATGCATACAACGTAACGACAAATCAAGATGGCACAGTATCATATGGC** |
| **P** | **GCTCGCCCGACACAAAAAAAGCCAAGCGAAACAAATGCATACAACGTAACAACACATGCAAATGGTCAAGTATCATATGGC** |
| **Q** | **GCTCGTCCAACACAAAACAAGGCAAGCAAAACAAACGCATATAACGTAACAACACATGCAAACGGTCAAGTATCATACGGA** |
| **R** | **GCTCGCCCAACACAAAATAAGCCATCAGAAACAAATGCATACAACGTAACAACACATGCAAATGGTCAAGTATCATATGGC** |
| **S** | **GCGAGACCAAGATTCAATAAGCCATCAGAAACAAACGCATATAACGTAACAACACATGCAAACGGTCAAGTATCATACGGA** |
| **T** | **GCTCGTCCGACATACAACAAGCCAAGCAAAACAAATGCATATAACGTAACAACACATGCAAATGGTCAAGTATCATACGGA** |
