# Supplementary figures and images for: Genetic Diversity of Methicillin‐Resistant Staphylococcus aureus Isolates From Two Tertiary Care Hospitals in Sulaymaniyah, Iraq, Characterized by spa Typing, Coagulase VNTR Sequencing, and REP‐PCR
Source: Int J Microbiol. 2026 Apr 20;2026:9366780. doi: 10.1155/ijm/9366780 (PMC13094366; doi:10.1155/ijm/9366780)

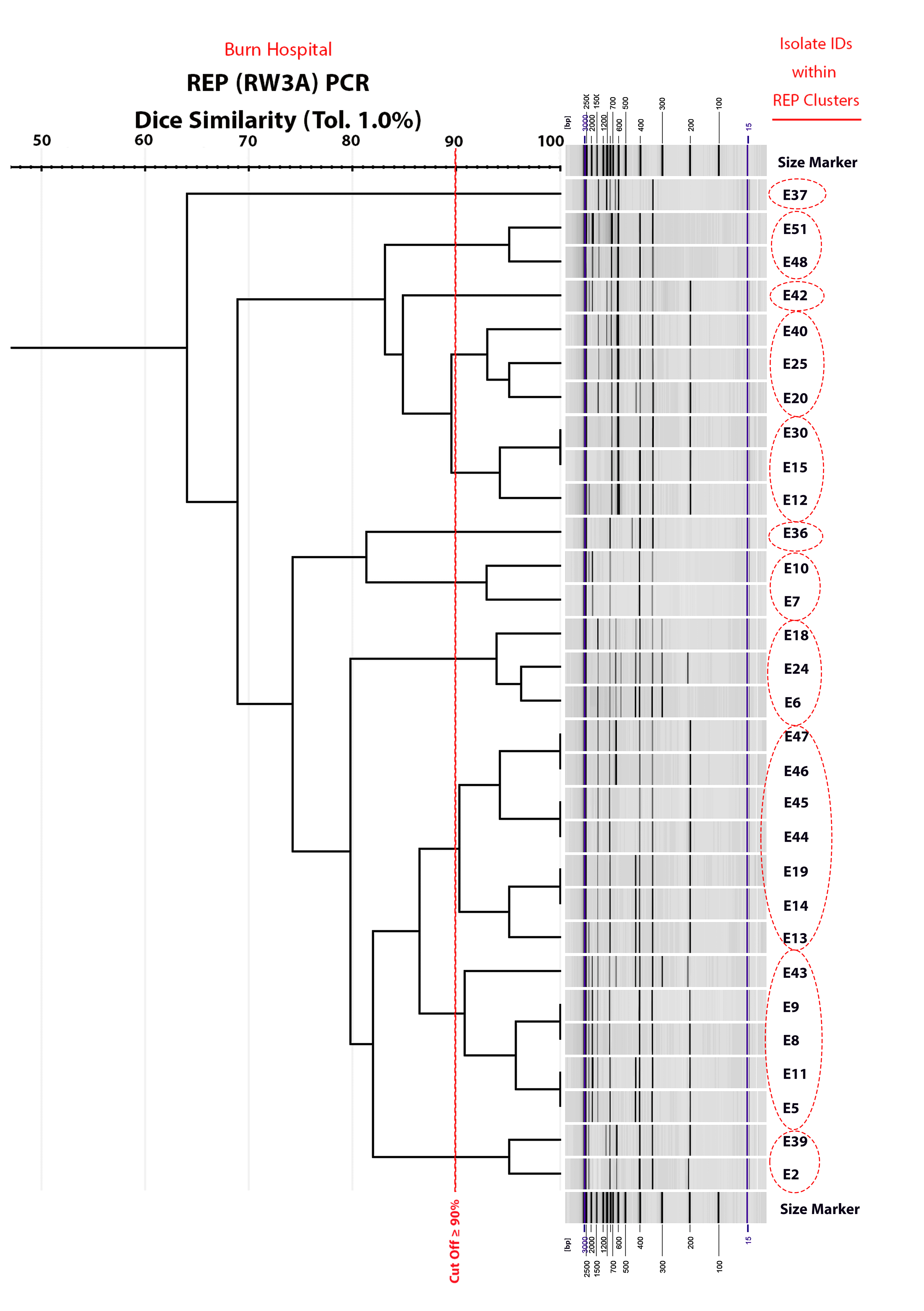

Supplement: Supplementary file 2 — Supporting Information 2 Supporting Figure S1: REP‐PCR (RW3A) fingerprinting dendrogram of MRSA isolates from the burn hospital (n = 30), showing clustering based on Dice similarity coefficients and UPGMA analysis. [file IJM-2026-9366780-s002.tif]

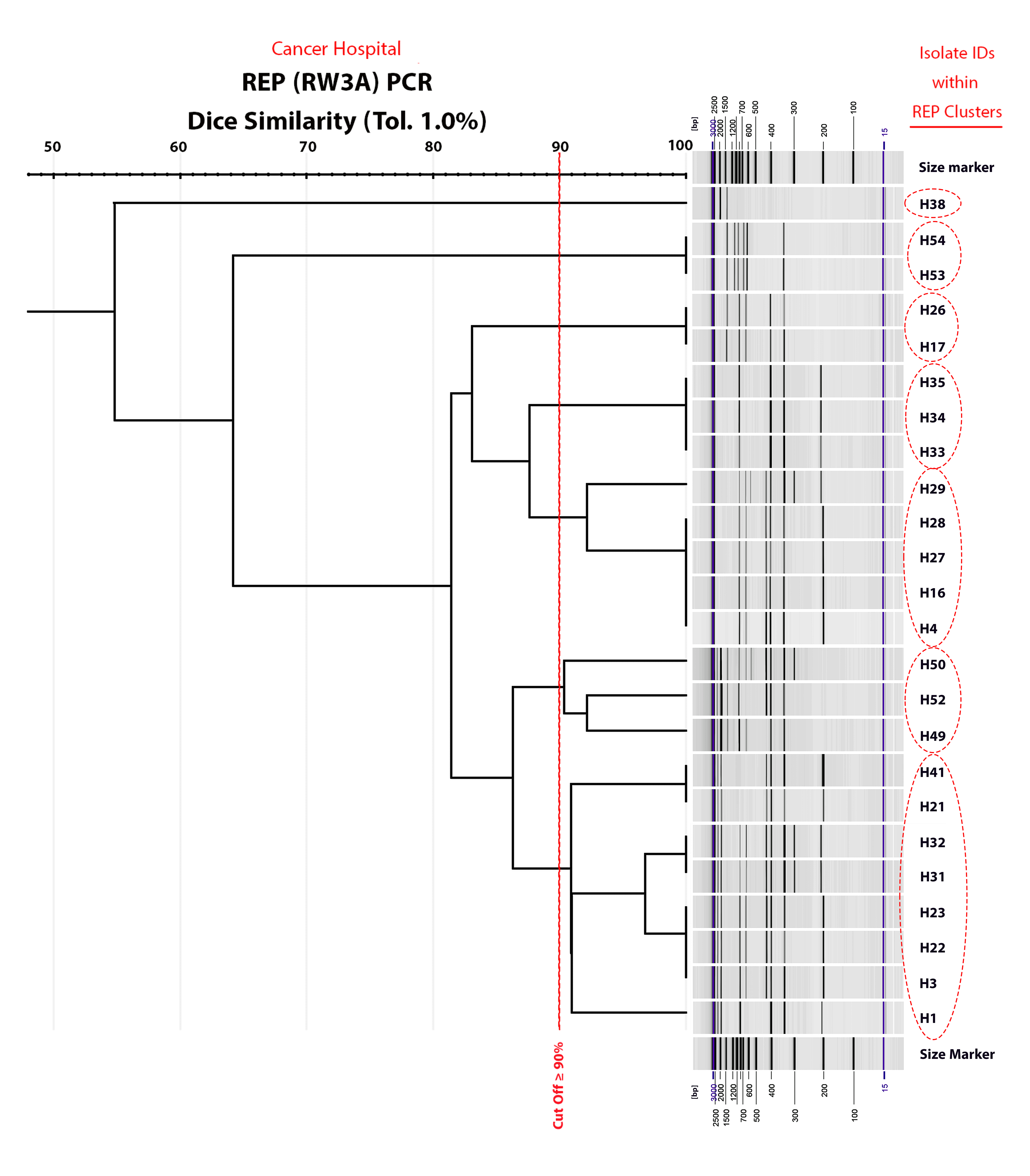

Supplement: Supplementary file 3 — Supporting Information 3 Supporting Figure S2: REP‐PCR (RW3A) fingerprinting dendrogram of MRSA isolates from the cancer hospital (n = 24), showing clustering based on Dice similarity coefficients and UPGMA analysis. [file IJM-2026-9366780-s003.tif]
